# Comment on “Complete Androgen Insensitivity Syndrome: Optimizing Diagnosis and Management”

**DOI:** 10.1155/2014/285715

**Published:** 2014-05-12

**Authors:** Antonio Balsamo, Federico Baronio, Marta Berra, Silvano Bertelloni, Franco D'Alberton, Giacinto Marrocco, Santiago Vallasciani

**Affiliations:** ^1^Department of Medical and Surgical Sciences, Pediatric Unit, Program of Endocrinology, University of Bologna, Italy; ^2^Department of Obstetrics and Gynecology, University of Bologna, Italy; ^3^Adolescent Medicine, Department of Obstetrics, Gynecology and Pediatrics, University Hospital, Pisa, Italy; ^4^Clinical Psychology Unit, Department of Pediatrics, University of Bologna, Italy; ^5^Pediatric Surgery, San Camillo-Forlanini Hospital, Roma, Italy; ^6^Pediatric Urology Unit, IRCCS Ca' Granda, Ospedale Maggiore Policlinico, Milan, Italy

We read with interest the paper of Pizzo et al. [1], confirming that adolescence is a key period for the diagnosis of 46,XY disorders of sex development (DSD) [2].

However, in our opinion, some points should be better addressed. Among these, we have the following.

1. The authors stated that the reported girl showed “normal intellectual function,” but this information had little relevance because mental function is not impaired in females with 46,XY DSD, reaching adolescence without any clinical suspicions. Indeed, this statement might be stigmatizing for these persons.
2. The statement that this adolescent was affected by hypergonadotropic hypogonadism (HH) is correct, according to the high LH and FSH values shown in Table 1 [1]. Very low levels of both 17*β*-estradiol and testosterone were also shown [1]. This endocrine pattern is not typical of late adolescent and young adult females with complete androgen insensitivity syndrome (CAIS) and intact testes. On the contrary, these persons show high/normal levels of both 17*β*-estradiol (for a person with a 46,XY karyotype) and testosterone [3–5]. In addition, adolescent/young adult women with CAIS did not show HH: LH is in high normal range or slightly elevated (due to the androgen resistance at central level), but FSH is in normal range (due to unaffected inhibin secretion from Sertoli cells) [3–5]. In addition, SHBG was reported within adult male range by Pizzo et al. [1], suggesting a normal sensitivity to the low levels of androgens in this girl [6]. Indeed, SHBG is within normal female range in women with CAIS, due to peripheral androgen resistance [5]. All these findings show poor agreement with the affirmed diagnosis of CAIS [1].
3. The clinical phenotype of the reported girl did not match with the phenotype of adolescents with CAIS; in fact, the latter shows normal breast development related to the relatively high normal estrogen levels and unopposed androgen action [3, 4, 7] and does not show hypotrophic breasts [1].
4. It is quite surprising that hormonal replacement therapy was started before any diagnosis was established.
5. Histological findings do not completely agree with the diagnosis of CAIS, in particular the absence of Leydig cells, which are abundant in adolescent females with CAIS and sometimes formed aggregates up to 2 mm in diameter [8, 9].
6. 46,XY karyotype, female phenotype, and absence of mullerian derivatives may be present in several 46,XY DSD; they should be excluded before diagnosis of CAIS by optimal clinical, endocrine, and genetic investigations [10]. For example, the testosterone/Δ_4_-androstenedione ratio in the adolescent reported by Pizzo et al. [1] is very low (0.13; normal values >0.8 [11]). Thus, diagnoses of 17*β*-hydroxysteroid-dehydrogenase deficiency type 3 or 46,XY gonadal dysgenesis [11, 12] must be considered in the diagnostic process.
7. Risk of gonadal cancer largely varies among 46,XY DSD. For example, in CAIS is very low at least during the first two decades of life [13–15]. Thus, delayed gonadal removal can be recommended to permit both full sexual development [15] and better bone health [16]. If diagnosis of CAIS is certain, surgery can be postponed—at least until the legal age at which the propositus can participate in decision making [1, 15, 17–19].
8. We are concerned and in complete disagreement with the decision to perform gonadectomy without full disclosure and assent of the adolescent [17–19].

In conclusion, some findings are poorly consistent with a diagnosis of CAIS, which should be confirmed by molecular analysis of androgen receptor gene [2, 7]. In our experience, more than 25% of the females referred to our departments with clinical/endocrine diagnosis of CAIS did not have this diagnosis confirmed by genetic analysis [20].

Clinical approach should be changed according to the new guidelines for management of persons with 46,XY DSD and directly involving the girl in the decision process [2, 18, 21]. We also stress that multidisciplinary team evaluation in tertiary centers with documented experience in this field must be guaranteed to each person with 46,XY DSD for optimal holistic management [21], especially before performing irreversible surgical procedures.
